# The function of Golgi apparatus in LRRK2-associated Parkinson’s disease

**DOI:** 10.3389/fnmol.2023.1097633

**Published:** 2023-02-21

**Authors:** Yonghang Wei, Maher un Nisa Awan, Liping Bai, Jie Bai

**Affiliations:** Laboratory of Molecular Neurobiology, Medical School, Kunming University of Science and Technology, Kunming, China

**Keywords:** Parkinson’s, LRRK2, Rab29, VPS52, PKC, Golgi apparatus

## Abstract

Parkinson’s disease (PD) is a chronic neurodegenerative disease associated with the intracellular organelles. Leucine-rich repeat kinase 2 (LRRK2) is a large multi-structural domain protein, and mutation in LRRK2 is associated with PD. LRRK2 regulates intracellular vesicle transport and function of organelles, including Golgi and lysosome. LRRK2 phosphorylates a group of Rab GTPases, including Rab29, Rab8, and Rab10. Rab29 acts in a common pathway with LRRK2. Rab29 has been shown to recruit LRRK2 to the Golgi complex (GC) to stimulate LRRK2 activity and alter the Golgi apparatus (GA). Interaction between LRRK2 and Vacuolar protein sorting protein 52 (VPS52), a subunit of the Golgi-associated retrograde protein (GARP) complex, mediates the function of intracellular soma trans-Golgi network (TGN) transport. VPS52 also interacts with Rab29. Knockdown of VPS52 leads to the loss of LRRK2/Rab29 transported to the TGN. Rab29, LRRK2, and VPS52 work together to regulate functions of the GA, which is associated with PD. We highlight recent advances in the roles of LRRK2, Rabs, VPS52, and other molecules, such as Cyclin-dependent kinase 5 (CDK5) and protein kinase C (PKC) in the GA, and discuss their possible association with the pathological mechanisms of PD.

## Introduction

### Parkinson’s disease (PD) and the Golgi apparatus (GA)

Parkinson’s disease (PD) is the second most common neurodegenerative disease in the world. The incidence of PD is expected to rise in the future (Tolosa et al., 2021). PD is characterized by motor and non-motor impairments associated with dopamine deficiency and others. The pathogenesis of PD is complicated and still unknown. Environmental and genetic factors cause mitochondrial dysfunction, protein aggregation, oxidative stress, impairment of autophagy, and neuroinflammation in PD (Simon et al., 2020). The mechanisms and the most promising strategies for developing effective therapies for PD are needed (Erb and Moore, 2020).

The basic structure of Golgi complex (GC) includes: inner Golgi, cis-Golgi, and trans-Golgi network (TGN) (Nakano, 2022). PD is related to apoptosis, DNA fragments and pro-apoptotic molecules increased in the dense dopaminergic neuronal regions of the substantia nigra (SN) of PD patients (Lev et al., 2003). A common feature of neurodegenerative diseases, including PD, is the fragmentation of GC (Gonatas et al., 2006; Fan et al., 2008; Caracci et al., 2019; Martinez-Menarguez et al., 2019). Early postmortem examinations of PD samples showed a high level of fragmentation of GC in some dopaminergic neurons (Fujita et al., 2006). Alpha-synuclein (α-syn) has been implicated in the pathogenesis of PD, however, the exact mechanism is not known (Du et al., 2020; Shahnawaz et al., 2020). The pathological hallmark of PD is mainly the aggregation of intracellular α-syn (Wakabayashi et al., 2007). The damage of the Golgi apparatus (GA) triggers the aggregation of α-syn and results in the formation of inclusions (Rendon et al., 2013). Aggregation of α-syn inhibits Golgi-related transport and leads to the accumulation of toxic substances causing oxidative stress and cell death (Lashuel and Hirling, 2006). Protein processing and transport in the GA are involved in apoptosis, in which the structure and function of the GA are disrupted (van Dis et al., 2014). Thus, the GA is associated with PD. In central nervous system (CNS) pathological conditions, the GC fragmentation has been observed in the early stages of apoptosis, therefore, the GC fragmentation is unlikely to be the result of apoptotic cell death (Liazoghli et al., 2005; van Dis et al., 2014). However, the exact mechanism and function of the GA on the apoptotic process are not clear (Caracci et al., 2019). This review focuses on these studies illustrated the relationship between GA and PD.

### Leucine-rich repeat kinase 2 (LRRK2) and Rabs

Leucine-rich repeat kinase 2 (LRRK2) is a large, multi-domain protein with kinase and GTPase domains (Nguyen and Moore, 2017). Mutations in the LRRK2 gene can present in patients with autosomal dominant PD and be associated with developing of sporadic PD (Tolosa et al., 2020). LRRK2 mutations account for approximately 1% of patients with sporadic PD and 5% of patients with familial PD, which suggests that LRRK2 is one of the commonly mutated genes associated with PD (Simpson et al., 2022).

The molecular mechanism of LRRK2 associated PD is unclear, however, LRRK2 in different cell types or models regulates TGN and lysosomal function, vesicle endocytosis and transport, and autophagy (Erb and Moore, 2020). Perez-Carrion et al. (2022) found that LRRK2 is related to the accumulation of α-syn. However, Henderson et al. (2019) showed that there was no necessary association between α-syn pathogenesis and LRRK2 in the PD mouse model. Therefore, this question needs to be further studied in the future. LRRK2 plays a crucial role in the GA. LRRK2 mutants affect the GA integrity and vesicle trafficking (Stafa et al., 2012; Beilina et al., 2014; Fujimoto et al., 2018; Purlyte et al., 2018). It has been shown that the inactivation of LRRK2 leads to the Golgi fragmentation and disrupts vesicle trafficking in human kidney proximal tubular epithelial cells (Lanning et al., 2018), even affects the entire endosomal system, including endocytosis and autophagy (Piccoli and Volta, 2021). The GA is indispensable in the endosomal system, and its dysfunction affects organelles such as endosomal and lysosomal function, synaptic vesicle trafficking, and ultimately alters neuronal function and synaptic plasticity (Piccoli and Volta, 2021).

Rabs, a kind of small GTPases involved in intracellular vesicular transport are important molecular switches for vesicular transport, and play a regulatory role in membrane transport in eukaryotic cells (Xu et al., 2021). The Rab binds with Rab effectors through binding domain (RBD) and recruits effectors to subcellular compartments to exert their effects. Effectors are used to regulate vesicle formation, transport and fusion by using other domains (Waschbusch et al., 2021; Zhang et al., 2022).

**FIGURE 1:**
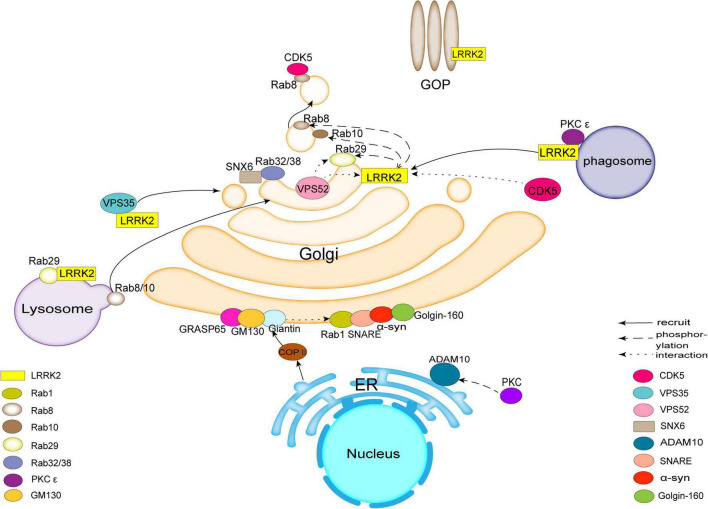
Leucine-rich repeat kinase 2 (LRRK2) acts as a hub to bind to molecules such as Rab family proteins, Vacuolar protein sorting protein (VPS), Golgi outposts (GOPs), Cyclin-dependent kinase 5 (CDK5), protein kinase C (PKC), synapse-associated protein 97 (SAP97), etc., affecting trans-Golgi network (TGN) membrane transport or Golgi morphology and ultimately inducing pathological features associated with Parkinson’s disease (PD). There are also molecules in the Golgi apparatus (GA), such as Golgin-160, Golgi matrix protein 130 (GM130), syntaxin 5, and others involved in related transport.

LRRK2 can directly phosphorylate Rabs (Steger et al., 2016). LRRK2 overexpression phosphorylates 14 Rabs, but only 10 Rabs are endogenous LRRK2 substrates (Rab3A/B/C/D, Rab8A/B, Rab10, Rab12, Rab35, and Rab43) (Ito et al., 2016; Steger et al., 2017; Thirstrup et al., 2017; Pfeffer, 2022). Rab8, Rab10, and Rab29 (also known as Rab7L1) interact with LRRK2. Rab8a, Rab8b, and Rab10 act downstream of LRRK2, while Rab29 appears to act upstream of LRRK2 (Eguchi et al., 2018; Liu et al., 2018; Kuwahara and Iwatsubo, 2020; Figure 1). LRRK2 also phosphorylates Rab29 (Fujimoto et al., 2018), and is inversely activated by GTP-bound Rab29, which suggest that there is reciprocal regulation between LRRK2 and Rabs (Liu et al., 2018). Rab29 recruited mutant LRRK2 to TGN or TGN-derived vesicles when these molecules were overexpressed (MacLeod et al., 2013; Beilina et al., 2014). LRRK2 activation and localization are regulated by Rab29 (Purlyte et al., 2018). Rab29 also recruits LRRK2 to lysosomes under lysosomal stress (Eguchi et al., 2018). LRRK2 phosphorylates and recruits Rab8 and Rab10 (Kuwahara and Iwatsubo, 2020). Rab10 phosphorylation at threonine 73 (pRab10 Thr73) by LRRK2 is regulated by activity and cellular localization of LRRK2 (Turski et al., 2022). Rab8 and Rab10 phosphorylated by LRRK2 accumulate around the centrosome and result in insufficient centrosome cohesion (Lara Ordonez et al., 2019). The pathogenic LRRK2 causes centrosome defects is independent of Rab29 or Golgi integrity. In contrast, in the presence of Rab29, centrosome changes affected by wild-type LRRK2 depend on Golgi integrity (Madero-Perez et al., 2018).

The common mutations of LRRK2 related to PD are G2019S, I2020T, and ROC-COR domains (R1441C/G/H, Y1699C) (Rudenko and Cookson, 2014; Cookson, 2015). *In vivo*, several familial PD mutations enhanced LRRK2 autophosphorylation on Ser1292 including R1441G/C, N1437H, G2019S, and I2020T, which suggest that Ser1292 autophosphorylation may be an indicator of LRRK2 kinase activity (Sheng et al., 2012). Steger et al. (2016) identified a subset of Rab GTPases as key LRRK2 substrates in cells, such as Rab10 and Rab12. The phosphorylation of a group of LRRK2 substrates are increased by all pathogenic LRRK2 mutants in intact cells, but only the G2019S mutant increases the phosphorylation *in vitro* (Blanca Ramirez et al., 2017). 14-3-3 is involved in the regulation of PD (Berg et al., 2003; Chen et al., 2003). LRRK2 mutations, such as R1441C, R1441G, R1441H, Y1699C, and I2020T, inhibited phosphorylation of LRRK2’s two conserved residues (Ser910 and Ser935) and disrupted the interaction between LRRK2 and 14-3-3, finally resulted in the accumulation of LRRK2 (Nichols et al., 2010).

Rab32, Rab38, and Rab29 have been shown to regulate the subcellular localization of LRRK2 through direct interactions (Waschbusch et al., 2014; Figure 1). Purlyte et al. (2018) showed that Rab29 binds to the ankyrin domain of LRRK2, and conserved residues in the domain enable Rab29 to mediate Golgi recruitment and kinase activation. However, McGrath et al. (2021) found the interaction between Rab29 and the ARM of LRRK2, one binding site followed by ankyrin repeats. Further research is needed. Rab32 interacts directly with sorting nexin 6 (SNX6), a subunit of the retromer (Waschbusch et al., 2019; Figure 1). Rab32/38 sorting nexins and retromer regulate signaling pathways on LRRK2 activation (Waschbusch et al., 2019). Missense mutation in Rab32 is associated with PD (Waschbusch et al., 2014). The localization of the mannose-6-phosphate receptor is regulated to the TGN by Rab32 and SNX6/retromer which are associated with Golgi trafficking (Waschbusch et al., 2019).

### Golgi-associated retrograde protein (GARP)

Vacuolar protein sorting protein 52 (VPS52) is a subunit of Golgi-associated retrograde protein (GARP) complex (Beilina et al., 2020). Beilina et al. (2020) found that the interaction between LRRK2 and VPS52 facilitated the interaction of GARP complex with Golgi SNAREs in TGN and promoted retrograde transport of TGN (Figure 1). Thus, the retrograde transport and subsequent transport pathway of TGN is regulated by the activity of LRRK2. VPS52 interacts with Rab29, and its knockdown results in a loss of LRRK2/Rab29 transport to the TGN. These results suggest that VPS52 plays a role in regulating LRRK2 and Rab29 transport to the TGN (Beilina et al., 2020).

### Retromer

Vacuolar protein sorting protein 35 (VPS35) (PARK17) is a molecule of retromer that selectively promotes endosomal-Golgi retrieval of transmembrane proteins. The retromer primarily selects and binds transmembrane protein cargo on the endosomal membrane to facilitate endosome-to-TGN or endosome-to-plasma membrane recycling. The autosomal dominant missense mutation Asp620Asn (D620N) in VPS35 is the only mutation in VPS35 that causes the late onset of PD (Vilarino-Guell et al., 2011; Zimprich et al., 2011; Williams et al., 2022). It has been shown that D620N VPS35 mutation increases the phosphorylation of LRRK2 (Mir et al., 2018). The interaction of the pathogenic G2019S LRRK2 mutation and D620N VPS35 enhances LRRK2 activity in SH-SY5Y cells (MacLeod et al., 2013). Wild-type LRRK2 activity was significantly reduced after CRISPR/CAS9 knockout of VPS35, whereas knockdown of VPS35 inhibits the kinase activity of LRRK2 (Mir et al., 2018). These studies suggest that VPS35 is upstream of LRRK2.

### Golgi outposts (GOPs)

The transport role of GA is related to common neuronal defects in neurological diseases, such as altered synaptic morphology, dendritic arborization and neuronal migration (Caracci et al., 2019). Among neurons the Golgi outposts (GOPs) are the important components of the dendritic secretory pathway, which contain shafts, branch points, and terminal branches (Pierce et al., 2001; Horton and Ehlers, 2003; Horton et al., 2005). Most GOPs are maintained in a stationary state, but some GOPs move toward the dendritic end (anterograde) or cell body (retrograde) (Lin et al., 2015). GOPs play a crucial role in synaptic connection (Caracci et al., 2019; Figure 1). LRRK2 is located at the dendritic site, regulates the dynamics of GOPs and even inhibits the movement of GOPs. Thus, LRRK2 plays an important role in regulating the localization of GOPs in neurons (Caracci et al., 2019). Lin et al. (2015) found that in *Drosophila melanogaster*, loss of function for dLRRK (*Drosophila* LRRK2) enhances cis-transport of GOPs, while overexpression of dLRRK inhibits cis-transport of Golgi. LRRK2 mutant G2019S promotes retrograde transport and increases the number and size of fixed GOPs located in Golgi vesicle branching sites in dendrites (Horton et al., 2005). This study shows that human LRRK2, similar to dLRRK which is crucial for GOPs regulation and contributes to PD development (Lin et al., 2015).

### Cyclin-dependent kinase 5 (CDK5)

Cyclin-dependent kinase 5 (CDK5) is a member of the cyclin-dependent kinase family and plays a key regulatory role in the cell cycle (Beaudette et al., 1993; Shah and Rossie, 2018). Dendritic length and synapses are influenced by CDK5 in dorsal striatal (DS) neurons (Zhou et al., 2022).

Cyclin-dependent kinase 5 is associated with neurodegenerative diseases, including PD (Cruz et al., 2003; Qu et al., 2007). Circuit impairment in the basal ganglia system results in PD, in which dopamine signaling in the striatum is negatively regulated by CDK5 (Shu et al., 2016). The LRRK2 R1628P mutation increases the binding affinity of LRRK2 to CDK5 and turns the adjacent amino acid residue serine S1627 of LRRK2 into a new phosphorylation site and activated by CDK5 (Shu et al., 2016).

Golgi fragments appear early in neurodegenerative diseases, however, the mechanism leading to fragmentation remains unclear (Gonatas et al., 2006; Nakagomi et al., 2008). The GA is affected by cell division cycle protein 2 (CDC2) kinase during mitosis. A study confirmed that inhibiting CDK5 using a persistent TAT-CDK5 inhibitory peptide (TAT-CIP) hindered Golgi division, suggesting that CDK5 plays a key role in Golgi division (Sun et al., 2008).

Rab8 regulates the transport of extracellular vesicles from the TGN to the peripheral membrane in the secretory pathway (Stenmark, 2009; Figure 1). G-protein-coupled receptor-activation-based (GRAB) is a guanine nucleotide exchange factor for Rab8 and a novel regulator of axonal growth. GRAB promotes the axonal membrane transport by mediating the interaction between Rab11 and Rab8 in neurons (Furusawa et al., 2017). GRAB is a substrate for the membrane-bound kinase CDK5. Thus, CDK5 can regulate neuronal function through regulating GRAB and membrane transport (Furusawa et al., 2017).

### Protein kinase C (PKC)

Golgi-associated protein kinase C (PKC) is composed of calcium- and phospholipid-dependent Ser/Thr protein kinases that mediate central cell signaling pathways and cause several neurological disorders such as PD (Rosse et al., 2010; Ohashi et al., 2017). The GA function is regulated by PKC and oxidative stress (Lenkavska et al., 2020). Oxidative stress has been suggested to play a key role in PD (Wei et al., 2018).

There are nine PKC genes in mammals, which are subdivided into three subfamilies: conventional PKC α, β, and γ, neo-PKC δ, ε, θ, η, and atypical PKC ε and ι (Steinberg, 2008). The Parkinsonian phenotype and disruption to dopamine signaling in the basal ganglia are found in AS/AGU (Albino Swiss/Anatomy Glasgow University) rats (Khojah et al., 2016). Knockout of PKC γ in animals, exhibits PD symptoms, such as loss of nigrostriatal dopaminergic neurons and movement disorder (Shirafuji et al., 2018). Golgi-associated PKC ε is linked and reached to the GA through the interaction with Golgi phosphatidylinositol 4-phosphate (PI4P) and diacylglycerol, and subsequently results in phagocytosis. When PKC ε is blocked from Golgi attachment, the level of PKC ε on the phagosome is reduced, then phagocytosis is reduced (D’Amico and Lennartz, 2018).

Zach et al. (2010) showed that LRRK2 interacted with PKC ε in mouse brain to alter neuronal structure and neuronal function through regulating oxidative stress. Sequence analysis identified several PKC phosphorylation sites contained in the LRRK2 protein, such as K/RXXS*/T* (Pearson and Kemp, 1991). LRRK2 is phosphorylated by recombinant PKC ε, however, PKC ε is not phosphorylated by LRRK2 (Figure 1). Thus, phosphorylated LRRK2 upon interaction between LRRK2 and PKC ε results in PD related pathological features through affecting the GA function.

### Synapse-associated protein 97 (SAP97)

Synapse-associated protein 97 (SAP97), a member of the membrane-associated guanylate kinase family, is a component of the stimulatory synaptic scaffold including postsynaptic density protein 95 (PSD95), PSD93, and SAP102 (Sans et al., 2001; Saraceno et al., 2014). It has been reported that the changes in SAP97 occurring in the human hippocampus and striatum are closely associated with PD (Di Maio et al., 2022). Studies of the postmortem hippocampus of patients with early PD by immunohistochemistry revealed a significant increase in SAP97 expression (Fourie et al., 2014). SAP97 expression was altered in the striatum of animal models of PD (Nash et al., 2005). In hippocampal pyramidal neurons GluR1, one of α-amino-3-hydroxy-5-methyl-4-isoxazolepropionic acid (AMPA) receptors, is located in the endoplasmic reticulum-cis-Golgi (ER-CG) (Sans et al., 2001). Phosphorylation of GluR1 reflects the activity of AMPA receptor, which has been shown to be associated with adverse reactions to dopaminergic treatment of PD (Ba et al., 2006). SAP97 interacts with the c-terminal PDZ domain of GluR1 to regulate its transport from the Golgi to the plasma membrane (Leonard et al., 1998). SAP97 is the only protein known to interact with the GluR1 PDZ-binding domain, and directly regulates the transport export from the ER (Sans et al., 2001; Waites et al., 2009). Saraceno et al. (2014) found that SAP97 transports A Disintegrin and Metalloproteinase 10 (ADAM10) from the dendritic GOPs to the synaptic membrane where ADAM10 and SAP97 formed a complex. SAP97 binds to PKC and affects PKC dependent cell migration (Saraceno et al., 2014). It has been shown that PKC activation positively regulates the interaction of ADAM10 with SAP97, and induces and facilitates ADAM10 transport from the ER to the postsynaptic membrane (Marcello et al., 2010). The phosphorylation of ADAM10 by PKC does not affect the ADAM10/SAP97 complex, only phosphorylation of SAP97 by PKC affects the formation of the complex (Figure 1). Phosphorylation of SAP97 T629 regulates the translocation of ADAM10 from the GOPs to the postsynaptic compartment, conversely, PKC dephosphorylation results in the accumulation of ADAM10 in the GOPs and synaptic reduction. When ADAM10 and SAP97 are uncoupled, ADAM10 triggered by PKC is not translocated from the Golgi precursor to the PSD and has not the effect on the sorting of proteins through the ER-somatic Golgi pathway (Saraceno et al., 2014).

### Golgin-160

Golgin A3, also known as Golgin-160, is involved in the transport of vesicles within the Golgi (Fritzler et al., 1993; Figure 1). In a yeast model experiment, overexpression of α-syn affected vesicular transport and resulted in transport inhibition, while Golgin-160 restored normal vesicular transport and decreased α-syn toxicity (Outeiro and Lindquist, 2003; Cooper et al., 2006). These studies suggest that Golgin-160 plays a role in PD through regulating transport pathway affected by α-syn.

### Golgi matrix protein 130 (GM130)

Golgi matrix protein 130 (GM130) located on the cis surface of the GA is the first identified matrix protein to tightly bind to the Golgi membrane to regulate the structure of the GA and plays an important role in maintaining the binding function of the GA (Marra et al., 2007). GM130 plays an important role in the development of the nervous system, however, it has not been studied well (Huang et al., 2021). GM130 C-end is combined with Golgi reassembly stacking protein 65 (GRASP65) and regulates GRASP65 position and stability (Puthenveedu et al., 2006; Figure 1). GM130 N-terminal binds to P115 and Giantin located on the vesicle membrane to form a complex consisting of GRASP65. GM130 and Giantin are involved in vesicle transport, such as transport of ER-budded Coat Protein complex II (COPII) vesicles to TGN (Alvarez et al., 1999; Marra et al., 2007; Sinka et al., 2008; Baba et al., 2018; Figure 1). GM130 inhibition leads to the accumulation of vesicle membranes and blocks the ER-Golgi transport pathway (Alvarez et al., 2001). It has been claimed that α-syn accumulates and binds abnormally to GM130 disrupting ER-Golgi transport. Rab1a, a mediator of vesicular transport, restores Golgi structure, improves hydrolase activity and reduces pathological α-syn expression in neurons (Mazzulli et al., 2016).

### Syntaxin 5

Syntaxin 5, a component of the soluble N-ethylmaleimide-sensitive factor attachment protein receptors (SNAREs) complex, is associated with ER-Golgi and intra-Golgi transport, maintains Golgi morphology and transport pathway. Syntaxin 5 interacts directly with the A53T mutant of α-syn (Thayanidhi et al., 2010). Binding of α-syn and SNARE forms a complex and mediates membrane fusion and synaptic vesicle release (Huang et al., 2019). Overexpression of Rab1 improves the endoplasmic reticulum to Golgi transport pathway that is inhibited by the toxic effect of α-syn (Cooper et al., 2006; Figure 1). α-syn also interacts directly with proteins involved in the maintenance of the Golgi interaction, such as SNARE (Thayanidhi et al., 2010). Therefore, Rab1 and SNARE are linked to the Golgi breakdown seen in PD and crucial for treating PD (Tomas et al., 2021).

## Conclusion

LRRK2 is a hub that binds to Rab family proteins, VPS, GOP, CDK5, PKC, SAP97, and other molecules that affect TGN membrane transport or Golgi structure. The GA damage triggers aggregation of α-syn that inhibits Golgi-associated transport and leads to the accumulation of toxic substances that cause oxidative stress and cell death. Autopsy in PD samples showed a high level of GC fragmentation, which suggests there is a close association between the GA and PD. LRRK2 also binds to related molecules in organelles such as mitochondria, endoplasmic reticulum, and lysosomes. There is an inseparable relationship between the GA and these organelles.

## Data availability statement

The datasets generated during and/or analyzed during the current study are available from the corresponding author on reasonable request.

## Author contributions

YW wrote the review. LB provided discussion. MA revised the review. JB instructed and re-revised the review. All authors contributed to the article and approved the submitted version.
